# 105 A Review of Regional Blocks During Enzymatic Debridement

**DOI:** 10.1093/jbcr/iraf019.105

**Published:** 2025-04-01

**Authors:** Zoey Chasen, Justin Suarez, Alexander Kurjatko

**Affiliations:** University of Iowa Burn Center; University of Iowa Health Care; University of Iowa Health Care

## Abstract

**Introduction:**

Anacaulase is a mixture of enzymes used for breakdown of eschars in patients with deep partial-thickness or full-thickness burns up to 20% of the body’s surface area. While anacaulase is an attractive therapy due to its ability to debride eschar, its use can result in significant pain. A European consensus guideline recommends regional anesthesia of an isolated extremity undergoing enzymatic debridement. No such consensus has been reached in North American societies. Pain strategies vary, but often include opioids with adjunct multimodal pain regimens. Our practice recently has utilized regional blocks prior to application of anacaulase. The purpose of this study is to determine whether patients receiving anacaulase therapy experienced improvement in pain with the addition of regional blocks to multimodal pain therapy.

**Methods:**

This is a single-center retrospective comparative study evaluating all patients who received anacaulase from July 31, 2016 to July 31, 2024. Chart review was performed to determine average pain scores and morphine milliequivalent requirements (MME) from the time of anacaulase administration to four hours after administration and receipt of adjunctive therapies for pain and sedation (including acetaminophen, dexmedetomidine, ketamine, midazolam, gabapentin, and ibuprofen). Continuous data was evaluated via Wilcoxon rank sum test and categorical data was evaluated via chi-square test of independence.

**Results:**

In the prespecified period, 47 patients received anacaulase and were included for analysis. Of the 47 patients, 24 (51.1%) underwent regional block. There were no statistically significant differences in baseline characteristics between groups, including average pain score four hours prior to anacaulase administration (4 vs 4.5, p=0.533). Pain scores in the four hours after anacaulase application were statistically significantly lower in patients that received nerve blocks and/or epidurals (4.5 vs 7, p=0.003). Additionally, MME four hours after anacaulase application were significantly lower (20 vs 33.6, p< 0.001) in the group that underwent regional block. There was no difference in requirement of adjunct medications except for an increase in dexmedetomidine requirement in the group that did not receive regional blocks (0% vs 78.3%, p < 0.001).

**Conclusions:**

Our data suggest that regional blocks were associated with lower pain scores and MME compared to multimodal pain therapy for burn patients receiving anacaulase therapy. Regional blocks may be an effective strategy in this population to reduce pain and opioid consumption.

**Applicability of Research to Practice:**

Further studies are needed to evaluate the effect of regional nerve blocks on patients undergoing enzymatic debridement with anacaulase.

**Funding for the Study:**

N/A

